# Supplementary material for: WordCommentsAnalyzer: A windows software tool for qualitative research
Source: F1000Res. 2018 Sep 4;7:536. Originally published 2018 May 3. [Version 2] doi: 10.12688/f1000research.14819.2 (PMC6137411; doi:10.12688/f1000research.14819.2)
Supplement: Supplementary file 3 [file f1000research-7-17614-s0002.tgz › b2a79496-4ada-47c4-9682-a99ce867836d.zip!/Eight Iranian Health Journals/Salmand_Iranian Journal of Ageing _0.docx]

© Salmand_Iranian Journal of Ageing . This work is licensed under CC BY-NC 4.0 (https://creativecommons.org/licenses/by-nc/4.0/).

Volume & Issue: دوره 13 - شماره 2

Title: آزمون‌های ارزیابی عملکردی تعادل در سالمندان

http://salmandj.uswr.ac.ir/article-1-1391-fa.html

اهداف اختلال تعادل یکی از اصلی‌ترین مشکلات سالمندان است که می‌تواند عملکرد روزانه را تحت تأثیر قرار دهد. درمان اختلالات تعادل هدف اصلی متخصصان این زمینه است. بنابراین، هدف این تحقیق مشخص کردن معتبرترین آزمون‌های عملکردی ارزیابی تعادل سالمندان برای امور بالینی است. مواد و روش ها جست‌وجوی الکترونیکی منابع اطلاعاتی PubMed، EMBASE، CINAHL، Magiran، IranMedex و IranDoc با استفاده از ترکیب کلیدواژه های «اندازه‌گیری، ارزیابی، اندازه‌گیری نتایج یا آزمون اندازه‌گیری»؛ «تعادل، موازنه، کنترل پاسچر، نشستن یا ایستادن»؛ «سالمند یا بازنشسته» و «سقوط، خطر سقوط، پیش‌بینی سقوط یا اختلالات تعادل»، و جست‌وجوی دستی و استفاده از منابع مقالات انجام شد. ضوابط ورود شامل هدف بیان‌شده ارزیابی تعادل، ارزیابی حداقل یک ویژگی روایی و پایایی، داشتن برنامه و ضوابط ساخت آزمون استاندارد بود که به انگلیسی یا فارسی منتظر شده بودند. یافته ها برای این پژوهش 41 آزمون تعادلی انتخاب شد. از بین آن‌ها آزمون‌های مقیاس تعادل برگ، زمان بلند شدن و رفتن، مقیاس تعادل پیشرفته فولرتون، آزمون غربالگری تعادل، آزمون رسیدن عملکردی، آزمون بالینی تعامل حسی در تعادل، ارزیابی اجرای جهت‌دار تحرک و ارزیابی راه رفتن عملکردی انتخاب شدند که ویژگی‌های مورد نظر این تحقیق را داشتند.  نتیجه گیری آزمون‌های انتخاب‌شده در این تحقیق مناسب‌ترین موارد برای استفاده در امور بالینی و پیشگیری از سقوط هستند.

Title: تحلیل محتوای مفاهیم و تصاویر سالمندی از بعد جسمانی در کتاب‌های درسی دوره ابتدایی ایران

http://salmandj.uswr.ac.ir/article-1-1188-fa.html

اهداف هدف از پژوهش حاضر، تحلیل محتوای مفاهیم و تصاویر سالمندی در کتاب‌های درسی دوره ابتدایی بعد از انقلاب اسلامی از این نظر بوده است که این کتاب‌ها از نظر جسمانی چه دیدی به سالمندی داشته‌اند. مواد و روش ها پژوهش حاضر پژوهشی توصیفی است که با روش تحلیل محتوا انجام شده است. جامعه آماری، تمامی کتاب‌های درسی دوره ابتدایی بعد از انقلاب است که از میان آن‌ها کتاب‌های سال‌های 1358، 1365، 1375، 1385 و 1394 انتخاب شده‌اند. دلیل انتخاب سال‌های یادشده این بود که این سال‌ها مقاطعی بودند که کتاب‌ها تغییر کرده‌اند. در این پژوهش واحد زمینه هر متن درس و واحد تحلیل مضامین و تصاویر مرتبط با سالمندی بوده است. در ضمن این مقاله در کمیته اخلاق دانشگاه علوم بهزیستی و توانبخشی به تأیید رسیده است. یافته ها نتایج این پژوهش مشخص کرد در کتاب‌های بررسی‌شده بیشتر سالمندان به صورت افرادی با چهره دارای چین‌وچروک (52/1درصد)، با موی خاکستری یا سفید (44/4درصد)، دارای قامتی صاف (57درصد) و با توان جسمانی زیاد (43درصد) تصویر شده‌اند. افرادی که عموماً از نظر جسمانی (72/5درصد) مستقل و سالم (73/2درصد) هستند. در این کتاب‌ها سالمندان عموماً (64/1 درصد) بدون استفاده از هیچ‌یک از وسایل کمکی تصویر شده‌اند و در مواردی که سالمندان با وسایل کمکی به تصویر کشیده شده‌اند، وسیله استفاده‌شده (14/1 درصد) عینک بوده است. نتیجه گیری نتایج نشان داد در کل تصویری طبیعی از وضعیت جسمانی سالمندان در کتاب‌های درسی ارائه شده است. به بیان دیگر تصویری که از سالمندان در کتاب‌های درسی ارائه شده است، تصویری عموماً متناسب با وضعیت جسمانی افراد در دوره سالمندی است. جالب آنکه در سال‌های اولیه بعد از انقلاب در مقایسه با سال‌های اخیر، تصویر جوان‌تری از سالمندان ارائه شده است که با توجه به روند افزایش امید به زندگی در دهه اخیر طبیعی و با واقعیت موجود سالمندی نیز متناسب است.

Title: ویژگی‌های روان‌سنجی نسخه فارسی پرسش‌نامه نیازسنجی کمبرول در سالمندان ایرانی مبتلا به اختلال روان

http://salmandj.uswr.ac.ir/article-1-1213-fa.html

اهداف ارزیابی و شناخت نیازهای برآورده‌شده و نشده سالمندان و برطرف‌کردن نیازهای برآورده‌نشده آن‌ها می‌تواند کیفیت زندگی سالمندان را افزایش دهد. برای استفاده از هر ابزار اندازه‌گیری اعتبار و پایایی آن ضروری است. این مطالعه با هدف بررسی تکرارپذیری در دفعات آزمون، اعتبار سازه و اعتبار معیار نسخه فارسی پرسش‌نامه نیازسنجی کمبرول در سالمندان ایرانی مبتلا به آسیب سلامت روان و مراقبان آن‌ها انجام شد. مواد و روش ها مطالعه حاضر از نوع توصیفی‌تحلیلی است که در سال 1394 روی 123 سالمند (61 نفر مرد و 62 نفر زن) مبتلا به آسیب سلامت روان (نمونه‌گیری آسان) با میانگین سنی (9/24)72 سال و 41 نفر از مراقبان غیررسمی آن‌ها انجام شد. تکرارپذیری در دفعات آزمون با انجام دو بار مصاحبه به فاصله حدوداً دو هفته و اعتبار معیار با سنجش همبستگی بین نمرات پرسش‌نامه کمبرول با پرسش‌نامه های 36 سؤالی کیفیت زندگی، شاه مقیاس شاخص بارتل و پرسش‌نامه 12 سؤالی بررسی سلامت عمومی بررسی شد. اعتبار سازه با تشکیل ماتریس همبستگی بین همه دو بخش پرسش‌نامه بررسی شد. تحلیل آماری داده ها با استفاد از نرم‌افزارSPSS19  انجام شد. یافته ها نتایج ضریب توافق کاپا در بررسی تکرارپذیری سالمندان (0/1-48/00)، مراقبان غیررسمی آن‌ها (0/1-65/00) و ضریب همبستگی درون طبقه ای در سالمندان 951/0 و مراقبان غیررسمی آن‌ها 970/0 در سطح معنی‌داری 05/0 به دست آمد. در بررسی اعتبار معیار هم‌زمان، همبستگی اسپیرمن بین نمره کل کمبرول با نمره کامل پرسش‌نامه کیفیت زندگی (790/0-) و شاه مقیاس شاخص بارتل (788/0-) و همبستگی متوسط بین نمره نیازهای برآورده‌نشده کمبرول با نمره کامل پرسش‌نامه کیفیت زندگی (474/0-) و همچنین همبستگی زیاد بین نمره بخش استرس روانی مراقبان کمبرول با نمره کامل پرسش‌نامه بررسی سلامت عمومی (875/0) به دست آمد. نتایج اعتبار، سازه خوب پرسش‌نامه را نشان داد. نتیجه گیری نتایج تکرارپذیری در دفعات آزمون، اعتبار سازه و اعتبار معیار هم‌زمان خوب نسخه فارسی پرسش‌نامه کمبرول را نشان داد. پیشنهاد می‌شود در مطالعات دیگری نیز ویژگی‌های روان‌سنجی پرسش‌نامه کمبرول ارزیابی شود.

Title: بررسی معنای زندگی و بهزیستی روانشناختی، جوانان، بزرگسالان و سالمندان (مطالعه مقایسه‌ای از نظر سن)

http://salmandj.uswr.ac.ir/article-1-1570-fa.html

اهداف هدف از پژوهش حاضر بررسی معنای زندگی و بهزیستی روان‌شناختی در سه گروه جوانان، بزرگسالان و سالمندان است. مواد و روش ها در این پژوهش 215 نفر به صورت نمونه‌گیری غیرتصادفی هدفمند انتخاب شدند. این تعداد عبارت بودند از: سالمندان بین 65 تا 80 سال (72نفر، 33/5 درصد)، بزرگسالان بین 26 تا 46 سال (59 نفر، 27/4 درصد) و جوانان با محدوده سنی 17 تا 25سال (84 نفر، 39/1 درصد). این افراد با استفاده از پرسش‌نامه‌های معنای زندگی استگر و بهزیستی روان‌شناختی ریف بررسی شدند. یافته ها به منظور بررسی مقایسه گروه‌های سنی در مؤلفه‌های معنا و بهزیستی روان‌شناختی از تحلیل واریانس چندمتغیری(مانووا) استفاده شد. نتایج به‌دست‌آمده از تحلیل نشان می‌دهد بین گروه سنی 17 تا 25، 25 تا 46 و 65 تا 80 سال، از نظر مؤلفه‌های وجود معنا، خودپذیری، روابط مثبت با دیگران، رشد فردی و زندگی هدفمند، تفاوت معنی‌دار وجود دارد. بر این اساس، مؤلفه‌های ذکرشده در سالمندان میانگین بالاتری دارند. نتیجه گیری نتایج حاصل از این پژوهش نشان می‌دهد عامل سن می‌تواند موجب افزایش معنای زندگی و بهزیستی روان‌شناختی شود. بنابراین می‌توان دوران سالمندی را دوره بازنگری و ارزیابی خود دانست و آن را در ادامه راه زندگی، زمان رشد و شکوفایی و آرامش قلمداد کرد.

Title: اثربخشی آموزش مرور زندگی بر رضایت از زندگی و احساس انسجام زنان میانسال و سالمند مقیم خانه سالمندان شهر قزوین

http://salmandj.uswr.ac.ir/article-1-1229-fa.html

هدف: سالمندی دروان حساسی از زندگی بشر است و توجه به مسائل و نیازهای این مرحله یک ضرورت اجتماعی است.  پژوهش حاضر با هدف اثربخشی آموزش مرور زندگی بر رضایت از زندگی و احساس انسجام زنان میانسال و سالمند مقیم خانه سالمندان  شهر قزوین انجام شد.  مواد و روش: طرح پژوهش حاضر از نوع نیمه‌تجربی با پیش‌آزمون و پس‌آزمون با گروه کنترل بود. جامعه آماری را کلیه زنان سالمند 50-80 خانه سالمندان ساکن در شهر قزوین تشکیل می‌داد، نمونه آماری پژوهش شامل 30 آزمودنی بود که به شیوه نمونه‌گیری تصادفی ساده انتخاب و در دو گروه 15 نفره (آزمایش و کنترل)، به شیوه تصادفی قرار گرفتند. برای جمع‌آوری داده‌ها از پرسشنامه‌های رضایت از زندگی داینر (1985)، احساس انسجام فلستنبرگ و همکاران (2006) استفاده شد. ابتدا همه نمونه‌ها به پرسشنامه‌های مذکور پاسخ دادند. سپس برنامه مرور زندگی بر روی گروه آزمایش در 6 جلسه اجرا شد و در نهایت پس‌آزمون از گروه آزمایش و کنترل گرفته شد. جهت تجزیه و تحلیل داده‌ها از نرم‌افزار SPSS و آزمون‌ تحلیل کواریانس استفاده شد. یافته ها: میانگین و انحراف معیار سن گروه کنترل 88/2  ± 65 و گروه آزمایش 56/3  ± 67 بوده است. میانگین رضایت از زندگی گروه کنترل قبل از آموزش 86/17 اما پس آموزش 60/17 و میانگین رضایت از زندگی گروه آزمایش قبل از آموزش 90/17 و بعد آموزش 86/24 بود. همچنین میانگین احساس انسجام گروه کنترل قبل از آموزش 80/69 و بعد از آموزش 93/69 در حالیکه میانگین گروه آزمایش قبل از آموزش 46/73 اما بعد آموزش 46/84 بود. نتایج تحلیل کواریانس نشان داد که فرایند جلسات درمان مرور زندگی باعث افزایش میزان رضایت از زندگی و احساس انسجام سالمندان در گروه آزمایش نسبت به گروه کنترل شده است(001/0p<). نتیجه گیری: نتایج این پژوهش حاکی از تأثیر آموزش مرور زندگی بر میزان رضایت زندگی و احساس انسجام سالمندان است. لذا می تواند به عنوان رویکرد درمانی جایگزین یا روش مکمل در کنار درمان های موجود برای بهبود زندگی در میان سالمندان مورد توجه قرار بگیرد.

Title: بررسی و مقایسۀ تعادل و انعطاف‌پذیری مردان و زنان سالمند در دامنه سنی 60 تا 79 سال

http://salmandj.uswr.ac.ir/article-1-1240-fa.html

اهداف آمادگی عملکردی مفهومی است که توانایی سالمند را برای اجرای فعالیت‌های جسمانی زندگی روزمره با سهولت نسبی منعکس می‌کند. هدف از پژوهش حاضر بررسی و مقایسۀ تعادل و انعطاف‌پذیری مردان و زنان سالمند در دامنه سنی 60 تا 79 سال خرم‌آباد بود.  مواد و روش ها این مطالعۀ توصیفی‌مقایسه‌ای، 140سالمند را (35 مرد و 35 زن در دامنه سنی 60 تا 69 سال و 35 مرد و 35 زن در دامنه سنی 70 تا 79 سال) به طور در دسترس انتخاب کرد که داوطلبانه در مطالعه شرکت کردند. برای ارزیابی تعادل و انعطاف‌پذیری به ترتیب از آزمون‌های دسترسی عملکردی و نشستن و رساندن استفاده شد. برای سهولت اجرا، آزمون‌ها در منزل آزمودنی‌ها انجام شد. برای تجزیه‌وتحلیل داده‌ها از آزمون T مستقل استفاده و سطح معنی‌داری 05/0>P در نظر گرفته شد.  یافته ها یافته‌ها نشان داد بین تعادل و انعطاف‌پذیری زنان و مردان در دامنه‌های سنی 60 تا 69 سال و 70 تا 79 سال اختلاف معناداری وجود دارد (05/0>P)؛ بین تعادل و انعطاف‌پذیری مردان در دامنه سنی 60 تا 69 سال و 70 تا 79 سال اختلاف معناداری وجود دارد (05/0>P)؛ بین تعادل و انعطاف‌پذیری زنان در دامنه سنی 60 تا 69 سال و 70 تا 79 سال اختلاف معناداری وجود دارد (05/0>P) و از سن 60 تا 79 سالگی تعادل و انعطاف‌پذیری مردان و زنان کاهش پیش‌رونده را نشان می‌دهد و میزان کاهش در زنان بیشتر از مردان است (79/15- در برابر 55/14- درصد برای تعادل و 79/17- در برابر 63/12- درصد برای انعطاف‌پذیری). نتیجه گیری با توجه به نقش بارز تعادل و انعطاف‌پذیری در انجام بهتر فعالیت‌های روزمره و کاهش آن‌ها در مردان و زنان سالمند، تقویت این دو بعد از عوامل آمادگی جسمانی در سراسر طول عمر مهم به نظر میرسد و برای تقویت این عوامل مداخلات ورزشی چندمؤلفه‌ای می‌تواند بهترین گزینه باشد.

Title: مقایسۀ سرمایه اجتماعی خانواده‌های دارای سالمند و بدون سالمند قم

http://salmandj.uswr.ac.ir/article-1-1307-fa.html

اهداف هدف از این پژوهش مقایسه سرمایه اجتماعی خانوادگی بین دو گروه خانواده است: خانواده‌های دارای سالمند و بدون سالمند. برخی از اندیشمندان علوم اجتماعی زندگی خانوادگی را سنگ بنای سرمایه اجتماعی می‌دانند که ریشه برخی از نابسامانی‌های اجتماعی امروز جامعه در نبود یا کمبود آن است. از همین رو، تقویت و تحکیم خانواده، بازگشت اخلاق به درون خانواده و ارتقای پیوند بین اعضای آن باعث ارتقای سرمایه‌های مهم جامعه می‌شود. مواد و روش ها پژوهش حاضر روی نمونه‌های معتبر جامعه آماری به حجم 384 نفر از خانواده‌های مناطق چهارگانه شهر قم و با روش پیمایش، فن پرسش‌نامه و روش نمونه‌گیری خوشه‌ای و تصادفی انجام شد. ابتدا براساس ملاک‌های عرفی و اجتماعی، شهر به سه منطقه تقسیم شد و در مرحله بعد از هر منطقه یک خیابان به صورت تصادفی انتخاب شد. در نهایت، با توجه به تشابه حدودی اندازه جمعیت هر منطقه، از یک یا دو بلوک در مناطق انتخاب‌شده، روی 100 پاسخ‌گو به صورت تصادفی مطالعه شد. 92 درصد پاسخ‌گویان مرد و 8 درصدشان زن بودند. 50 درصد از پاسخ‌گویان 26تا 35ساله و 33درصد 36تا 45 ساله و 17درصد باقی‌مانده 45سال و بیشتر بودند. یافته ها نتایج حاکی از این است که بین سرمایه اجتماعی شناختی درون‌خانوادگی در دو گروه خانواده‌های دارای سالمند و بدون سالمند تفاوت معناداری وجود ندارد. اما بین سرمایه اجتماعی ساختاری درونی و بیرونی و شناختی بیرونی خانواده دو گروه تفاوت معناداری دیده می‌شود. با لحاظ متغیرهای زمینه‌ای مانند جنسیت، تحصیلات، سن، تعداد فرزندان و اشتغال نیز بین دو گروه خانواده‌ها، تفاوت معناداری به وجود نیامد.  نتیجه گیری حضور سالمند در خانواده باعث افزایش سرمایه اجتماعی خانوادگی و به‌تبع آن کاهش مسائل عدیده اخلاقی جامعه خواهد شد. دولت‌ها باید با حمایت از سالمندان و برطرف‌کردن نیازهای آن‌ها اعمّ از خدمات درمانی و هزینه‌های دارویی، حمایت‌های اقتصادی و غیره، شرایط را برای حضور سالمند در کنار فرزندان و افزایش سرمایه اجتماعی خانوادگی فراهم کنند.

Title: مقایسه نگرش سالمندان و مدیران نسبت به وضعیت شاخص های شهرهای دوستدار سالمند

http://salmandj.uswr.ac.ir/article-1-1318-fa.html

هدف: فرایند ایجاد محیط های دوستدار سالمند از چالش های کلیدی سیاست گزاران است. هدف از مطالعه حاضر مقایسه دیدگاه سالمندان و مدیران نسبت به وضعیت شاخص های شهرهای دوستدار سالمند بود. مواد و روش ها: دراین مطالعه توصیفی-تحلیلی، دیدگاه  379سالمند و 57 مدیر مقایسه شد.ابزار گرداوری داده ها پرسشنامه شاخص های شهرهای دوستدار سالمند سازمان بهداشت جهانی(2013)بود. داده ها با آمار توصیفی(میانگین، انحراف معیار، درصد فراوانی) و آزمون گاما با استفاده از  SPSS نسخه 21 تحلیل شد. یافته ها: از 379 سالمند مورد بررسی 9/64%(246 نفر) زن بودند. میانگین سنی سالمندان 25/8 ±31/68 (حداقل 60 و حداکثر 89 سال) بود. در 13 شاخص از 21 شاخص، اختلاف معنی داری بین دیدگاه سالمندان و مدیران وجود داشت.عمده ترین شاخص هایی که از دیدگاه سالمندان بد تا بسیار بد ارزیابی شد و اختلاف معنی داری با دیدگاه مدیران داشت شامل موارد زیر بود: وجود جایی برای دریافت اطلاعات، جهت رفع نگرانی های بهداشتی و نیازهای خدماتی سالمندان(57%سالمندان درمقابل14% مدیران )( 001/ 0=P)؛ وضعیت سیاست گزاری برای ساخت مسکن های جدید مطابق نیازهای سالمندان(57% سالمندان درمقابل 6/31% مدیران)(014/0P=). نتیجه گیری: تشریک مساعی بین مدیران سازمان های متولی سالمندی با سالمندان، جهت دست یابی به شاخص های شهرهای دوستدار سالمند ضروری به نظر می رسد.

Title: بررسی تأثیر رایحه‌درمانی عصاره گل سرخ بر درد مزمن اسکلتی‌عضلانی سالمندان کانون‌های بازنشستگی

http://salmandj.uswr.ac.ir/article-1-1374-fa.html

اهداف یکی از عمده‌ترین مشکلات سلامتی دوران سالمندی، دردهای مزمن است. یکی از روش‌های کاهش دردهای این دوران رایحه‌درمانی است. هدف از انجام این مطالعه، بررسی تأثیر استنشاق عصاره گل سرخ بر درد مزمن اسکلتی‌عضلانی سالمندان است. مواد و روش ها این پژوهش نوعی مطالعه کارآزمایی بالینی است. نمونه‌گیری به روش در دسترس با تخصیص تصادفی روی ۶۰ سالمند مبتلا به درد مزمن اسکلتی‌عضلانی عضو کانون‌های بازنشستگی شهر سبزوار در سال 1395 انجام شد. سالمندان به صورت تخصیص تصادفی در دو گروه مداخله (31 نفر) و کنترل (29 نفر) تقسیم شدند. درد با پرسش‌نامه درد مک‌گیل که پرسش‌نامه‌ای روا و پایا در مطالعات دیگر بوده است، قبل از مداخله به صورت مصاحبه و هفته‌ای یک‌بار در روز پنج‌شنبه حین مداخله به صورت تلفنی در دو گروه اندازه‌گیری شد. در گروه مداخله، استنشاق عصاره گل سرخ با دستمال کتانی آغشته به سه قطره عصاره از ساعت 9 شب تا 6 صبح در فاصله 25 سانتی‌متری، یک نوبت در شبانه روز به‌مدّت سه‌هفته انجام شد. در گروه کنترل روغن بادام شیرین با روش مشابه به کار برده شد. داده‌ها با استفاده از نرم‌افزار SPSS نسخه 20 و با سطح معناداری 50/0P≤ تحلیل شد. یافته ها یافته‌ها نشان داد استنشاق عصاره گل سرخ بر بعد حسی و عاطفی درد در زنان و مردان تأثیرگذار نبود. این در حالی است که استنشاق عصاره گل سرخ بر بعد حسی در مقایسه بین زنان و مردان تفاوت آماری معنادار دارد (P≤0/05)؛ اما بر بعد عاطفی بین زنان و مردان تفاوتی ندارد. نتیجه گیری مطالعه حاضر نشان داد استفاده از رایحه‌درمانی عصاره گل سرخ باعث کاهش بعد حسی دردهای اسکلتی‌عضلانی بین زنان و مردان می‌شود؛ اگرچه انجام تحقیقات بیشتر در این زمینه ضروری است. یافته‌های حاصل از این پژوهش می‌تواند  برای محققان، مسئولان و متولیان در برنامه‌ریزی و مدیریت مؤثر درد مزمن سودمند باشد.

Title: انتظارات سالمندان مقیم سرای سالمندان از فرزندان خود

http://salmandj.uswr.ac.ir/article-1-1396-fa.html

اهداف پژوهش حاضر یک مطالعه کیفی است که به هدف شناسایی و توصیف انتظارات سالمندان مقیم سرای سالمندان از فرزندان خود انجام شد. مواد و روش‌ها در این مطالعه با بهره‌گیری از روش پژوهش فرهنگ نگاری، تعداد 30 نفر از سالمندان مقیم آسایشگاه سالمندان شهرستان ملایر با روش نمونه‌گیری هدفمند از نوع مبتنی بر معیار انتخاب شدند. داده­های پژوهش از طریق مصاحبه نیمه­ساختار گردآوری و با روش تحلیل مضمونی مورد تحلیل قرار گرفت. یافته‌ها نتایج تحلیل داده­های پژوهش به شناسایی چهار دسته انتطارات اساسی تحت عناوین مراقبت جسمانی و تأمین نیازهای بهداشت فردی سالمندان؛ حمایت اجتماعی و احترام به شان و جایگاه سالمندان در خانواده؛ حمایت عاطفی و حفظ استقلال و حریم شخصی سالمندان؛ و حمایت مالی و تأمین هزینه‌های اساسی زندگی سالمندان منجر شد. در مجموع، نتایج این پژوهش از نقش حیاتی نهاد خانواده در برآورده نمودن انتظارات و دغدغه‌های سالمندان و به تبع آن بهبود کیفیت زندگی جسمانی و روانی آنان حمایت می­کند. نتیجه‌گیری اعضای خانواده و به ویژه فرزندان افراد سالمند می­توانند با ایجاد شرایط و فرصتهایی مناسب به سالمندان اطمینان دهند که هم در تامین هزینه‌های مالی و نیازهای اساسی زندگی حامی و پشتیبان آنها هستند و هم به استقلال و توانمندی­ آنان بعنوان یک سرمایه اجتماعی برای جامعه باور دارند.
